# EEG-Based Emotion Recognition Using an Improved Weighted Horizontal Visibility Graph

**DOI:** 10.3390/s21051870

**Published:** 2021-03-07

**Authors:** Tianjiao Kong, Jie Shao, Jiuyuan Hu, Xin Yang, Shiyiling Yang, Reza Malekian

**Affiliations:** 1 College of Electronic and Information Engineering, Nanjing University of Aeronautics and Astronautics, Nanjing 210016, China; kongtianjiao@nuaa.edu.cn (T.K.); shaojie@nuaa.edu.cn (J.S.); hujiuyuan@nuaa.edu.cn (J.H.); yangxin009@nuaa.edu.cn (X.Y.); yang410@nuaa.edu.cn (S.Y.); 2 Key Laboratory of Underwater Acoustic Signal Processing, Ministry of Education, Southeast University, Nanjing 210096, China; 3 Department of Computer Science and Media Technology, Malmö University, 20506 Malmö, Sweden; 4 Internet of Things and People Research Center, Malmö University, 20506 Malmö, Sweden

**Keywords:** emotion recognition, EEG, directed weighted horizontal visibility graph, feature fusion

## Abstract

Emotion recognition, as a challenging and active research area, has received considerable awareness in recent years. In this study, an attempt was made to extract complex network features from electroencephalogram (EEG) signals for emotion recognition. We proposed a novel method of constructing forward weighted horizontal visibility graphs (FWHVG) and backward weighted horizontal visibility graphs (BWHVG) based on angle measurement. The two types of complex networks were used to extract network features. Then, the two feature matrices were fused into a single feature matrix to classify EEG signals. The average emotion recognition accuracies based on complex network features of proposed method in the valence and arousal dimension were 97.53% and 97.75%. The proposed method achieved classification accuracies of 98.12% and 98.06% for valence and arousal when combined with time-domain features.

## 1. Introduction

Emotion is the reflection of people’s psychological and physical expressions. It plays a crucial factor in decision-making, perception, and human-computer interaction (HCI) systems [1,2] Many studies based on emotion recognition have been conducted in the last few decades [3,4].

The methods of emotion recognition are usually divided into two categories. One is based on physiological signals, and the other is based on non-physiological signals. Non-physiological signals include facial expressions, speech signals, body movements, and so on [5,6]. Studies based on non-physiological signals have produced significant results. For example, virtual markers based on an optical flow algorithm were used to classify six facial emotions (happiness, sadness, anger, fear, disgust, and surprise) [7]. They achieved a maximum accuracy of 99.81% with the CNN classifier. Niu et al. [8] proposed fused features using the oriented fast and rotated brief (ORB) features and local binary patterns (LBP) features to classify seven facial emotions, in which the accuracy is 79.8%. However, emotion recognition through facial expressions or behavior analyzes is usually built on fake emotions, including photos of actors instead of faces expressing real emotional states. Datasets of real facial emotions are scarce. The expression and regulation of emotional cues are different in different countries [9]. It may affect the accuracy of the emotion classification. Therefore, research on recognizing emotions through physiological signals is being actively conducted.

Physiological signals are another approach for emotion recognition. Physiological signals include heart rate, functional magnetic resonance imaging, electromyography (EMG), electroencephalogram (EEG), and so on. Among them, emotion recognition based on EEG signals has great effects on detecting an emotion directly from brain activity [10]. For example, Thammasan et al. [11] proposed a continuous music emotion classification algorithm based on different features. Arnau et al. [12] used principal component analysis to selected features, achieving accuracies of 67.7% and 69.6% for valence and arousal. An emotion recognition model based on a mixture classification technique for physically challenged or immobilized people was proposed [13]. This model is an asymmetric distribution, which can help extract the EEG signals with a symmetric or asymmetric form.

In 2008, Lucasa et al. first proposed a visibility graph (VG) to map time series data into a complex network [14]. With the development of research, many improved VG algorithms were proposed. One of the modified visibility graphs, the horizontal visibility graph (HVG), has been submitted by Luque et al. [15]. HVG can represent the chaotic characteristics of EEG signals. Wang et al. used a limited penetrable visibility graph (LPVG) to analyze Alzheimer’s disease [16]. Zhu et al. proposed weighted horizontal visibility (WVG), which introduced the edge weight [17]. Recently, the visibility graph has been employed to analyze EEG signals. Bhaduri calculated the scale-freeness of the visibility graph of EEG data patterns varying from normal eye closed to epileptic [18]. This work provided the first quantitative analysis technique for the degree of fractality. Zhu et al. used difference visibility graphs to analyze and classify the EEG signals of sleep stages [19]. The accuracy of the six-state classification is 87.5%. Cai [20] et al. developed a novel multiplex LPHVG method to explore brain fatigue behavior. This method yields novel insights into the brain-behavior associated with fatigue driving. By employing the visibility graph algorithm, Bajestani et al. examined the EEG signals of patients with autism spectrum disorder (ADS) [21]. The ASD class can be discerned with an accuracy of 81.67%.

The effectiveness of complex network features in the classification of EEG signals has been demonstrated. However, few studies currently use complex network features for EEG-based emotion recognition. 

In this paper, a novel approach based on complex network features was presented for emotion recognition. Weighted complex networks based on new angle measurements were constructed. The innovation of this method is that we use a new weighted method to construct the directed visibility graph. On this basis, the fusion feature is used to improve the effectiveness of features. EEG signals were mapped into two complex weighted networks from different directions: forward weighted horizontal visibility graph (FWHVG) and backward weighted horizontal visibility graph (BWHVG). Two feature matrices were extracted from the two weighted complex networks. Then, the fusion feature of two feature matrices was used to classify the EEG signals. The fusion matrix was fed into three classifiers for training and testing.

## 2. Related Works

### 2.1. Emotion Classification of DEAP Dataset

Emotion datasets with different modalities were established by researchers, such as the DREAMER dataset, AMIGOS dataset, MAHNOB HCI dataset, and the DEAP dataset. The DEAP emotion database was used in this paper. There are many emotion recognition methods proposed based on the DEAP dataset. Lee et al. [22] proposed an emotion recognition model using a photoplethysmogram (PPG) signal of DEAP for the short recognition interval. Electrodermal activity (EDA) signals of the DEAP dataset were used to design a sensor for emotion recognition [23]. They achieved an accuracy of 85% for four class emotional states. Kim et al. [24] proposed a long short-term memory network based on EEG signals to consider changes in emotion over time. They performed the two-level and three-level classification experiments based on valence and arousal. The classification rates on two-level emotion recognition were 90.1% and 88.3% for valence and arousal 86.9% and 84.1% on three-level emotion recognition.

### 2.2. Emotion Recognition Based on Feature Extraction

Researchers have conducted studies to extract different features in the EEG-based emotion recognition task. Machine learning and deep learning techniques are applied to classify emotional states. Numerous attributes include power spectral density features (PSD), fractal dimension features (FD), entropy features, wavelet features, the differential asymmetry feature (DASM), the rational asymmetry feature (RASM), and the differential causality feature (DCAU) have been widely employed to characterize EEG [25,26]. Yin Y.Q et al. proposed a deep learning model fused graph convolutional neural network (GCNN) and long-short term memories neural networks (LSTM). Differential entropy was extracted to construct a feature cube as the input of the model. The average classification accuracies were 90.45% and 90.60% for valence and arousal on the DEAP dataset [27]. A dynamical graph convolutional neural network (DGCNN) using a graph to model the multichannel EEG features was proposed in [28]. Five kinds of features including differential entropy, DASM, RASM, PSD, and DCAU were investigated to evaluate the proposed method. The accuracy was 86.23% in valence and 84.54% in arousal on the DREAMER database. Goshvarpour et al. [29] extracted the approximate and detailed coefficients of the wavelet transform and calculated the second-order difference plot of the coefficients. The average classification rate was 80.24% on four different emotion classes. 

### 2.3. Emotion Models

A number of researchers have proposed different ways to express emotions, including the discrete emotion model, the dimensional emotion model, and other emotion models. In the discrete emotion model, researchers considered the theory of basic emotion, such as the Ekman emotion model [30] and the Panksepp emotion model [31]. There is a dispute about the number of basic emotions. Tuomas et al. believe that fear, anger, disgust, and happiness are the four basic emotions [32]. While Cowen et al. maintain that there are 27 basic emotions [33]. In the dimensional model, emotions are described by multiple dimensions, such as the circumplex model [34]. It’s a two-dimensional model of arousal and valence. When dominance is added, it can be extended to a 3D emotion model [35]. Many researchers have proposed different emotion models according to their different analytical perspectives, such as Ortony-Clore-Collins (OCC) model and hidden Markov model (HMM) [36,37].

## 3. Materials and Methods

### 3.1. Dataset

The DEAP dataset [38], a multimodal dataset created by Koelstra et al., is used in this paper. The dataset is publically available and many researchers have performed their analysis on it. The DEAP dataset consists of two parts, namely the online ratings and the participant ratings, contains 1280 multivariate biosignals, such as electroencephalogram, photoplethysmogram, electromyogram, and electrodermal activity. 

Table 1 describes the participant rating part. The participant ratings were acquired from 32 participants with an average age of 26.9 years, in which each subject watched 40 one-minute long music videos. After watching each video, participants assessed the videos at different levels ranging from 1 (low) to 9 (high). The emotional response includes five dimensions: valence, arousal, dominance, liking, and familiarity. Valence is an indicator of pleasantness. Arousal is a measure of the intensity of the emotion varying from unexcited to excited. Dominance represents the feeling of being in control of the emotion. Liking asks for participants’ liking of the video. Familiarity is the participants’ familiarity with each of the videos. Familiarity study participants’ tastes, not their feelings, on a scale of 1 to 5. For valence, arousal, dominance, and liking, the threshold is set as different values in different researches. The middle of the 9-point rating is used to generate two classes as used on the DEAP dataset. The label is low when the rating is less than 5, and the label is high when the rating is greater than or equal to 5.

Forty physiological channels were recorded for each participant, including 32 EEG channels and eight other peripheral channels. The data includes 60-s trial data and 3-s baseline data. 60-s trial data were used in this paper. The DEAP provide the preprocessed dataset. The data were down sampled to 128 Hz, and a bandpass frequency filter from 4.0–45.0 Hz was applied. Since emotions are generally described by arousal and valence, we only consider the two factors. 

### 3.2. Emotion Recognition Framework

**Figure 1:**
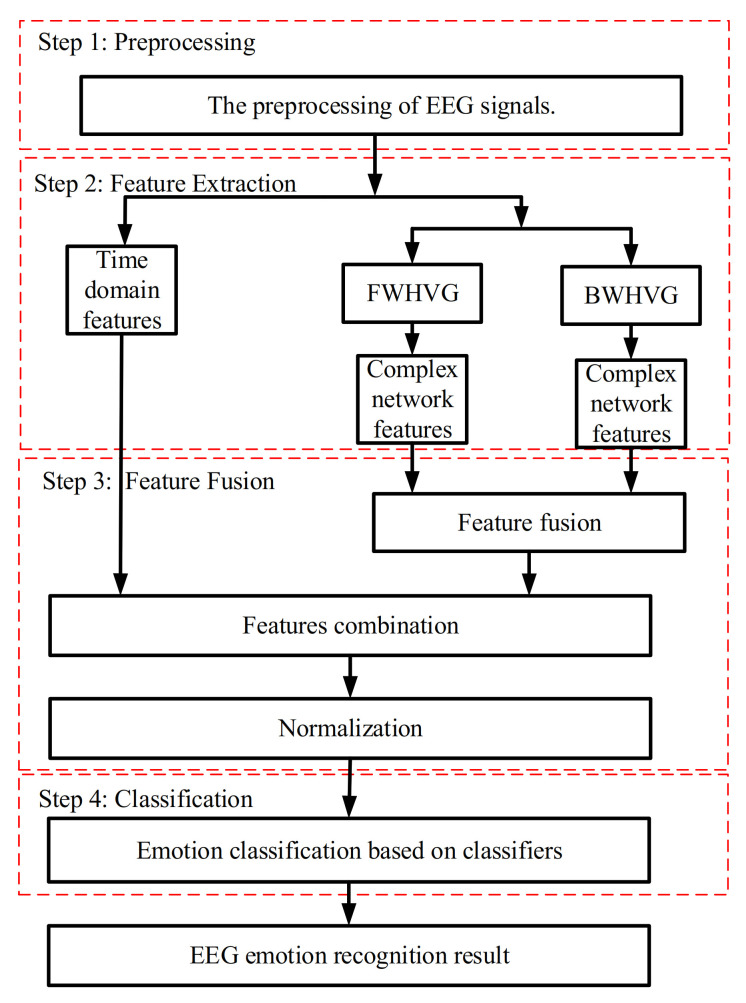
The block diagram of the proposed method for EEG emotion recognition.

The block diagram of the proposed method for EEG emotion recognition in this paper is shown in Figure 1. Thirty-two EEG channels are selected to classify emotional states in this paper. The procedure is divided into four steps, namely, preprocessing, feature extraction, feature fusion, and classification. The preprocessing includes data partitioning and channel selection. For the EEG signal data after preprocessing, time-domain features and network statistical properties are extracted. And then, the two types of features can be combined and normalized. Finally, three classifiers are used to train these features to obtain the results of emotion recognition. 

### 3.3. Visibility Graph Networks

#### 3.3.1. Horizontal Visibility Graph

VG algorithm can map time series to complex networks. For an EEG signal ${\left\{x\left(t\right)\right\}}_{t=1}^{N}$ with *N* data samples, each sample can be considered as a node of the graph represented in a histogram. The height of the histogram represents the value of the corresponding data node. There is a connection between two nodes if the top of two bars is visible. For any two nodes (*t_i_,x_i_*) and (*t_j_,x_j_*), the edge between *t_i_,* and *t_j_* is connected if any data node (*t_k_,x_k_*) between (*t_i_,x_i_*) and (*t_j_,xj*) fulfils the following criterion of convexity [14]:

(1) $$\frac{x_{i}-x_{k}}{t_{k}-t_{i}}>\frac{x_{i}-x_{j}}{t_{j}-t_{i}},t_{i}<t_{k}<t_{j},$$

HVG is a modification of the VG algorithm. In HVG, two data nodes (*t_i_,x_i_*) and (*t_j,_x_j_*) will have horizontal visibility if they fulfil Equation (2) [15]:

(2) $$x_{i},x_{j}>x_{k},\;t_{i}<t_{k}<t_{j},$$

where (*t_k_,x_k_*) is a data node between (*t_i_,x_i_*) and (*t_j_,x_j_*).

**Figure 2:**
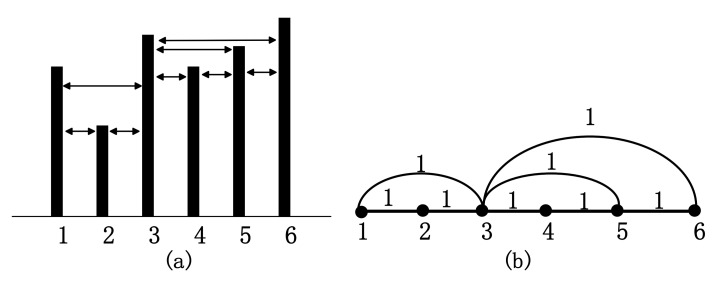
Horizontal visibility graph of a time series. (**a**) The histogram of time series; (**b**) Its corresponding HVG.

The complex network can be expressed by an adjacent matrix $A={\left(a_{ij}\right)}_{N\times N}$. If *t_i_* and *t_j_* are connected, *a_ij_* = 1, otherwise *a_ij_* = 0, as shown in Figure 2.

#### 3.3.2. Directed Weighted Horizontal Visibility Graph

**Figure 3:**
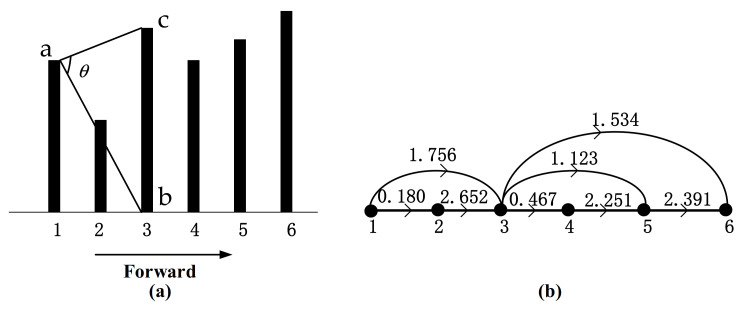
Graphical illustration of FWHVG of the time series. (**a**) Angle measurement of FWHVG; (**b**) Corresponding FWHVG of the time series.

HVG with edge weight is known as the weighted horizontal visibility graph (WHVG), where the link between two nodes are not binary values (0 and 1). There are two commonly used edge weights at present, namely distance [39] and radian function [40]. We proposed a novel directed weighted horizontal visibility graph (DWHVG). The edge weight is related to visibility angle measurement. The weighted complex network can be expressed by a weight matrix $W=\;{\left(w_{ij}\right)}_{NxN}$. The edge weight *w_ij_* is the angle between nodes *i* and *j*. It can be described as follows: if nodes *i* and *j* is visible, the connection of the vertex *i* and vertex *j* is called *ab*, and the connection of the vertex *i* and bottom *j* is called *ac*. The edge weight *w_ij_* is the angle between *ab* and *ac*, as shown in Figure 3a. Equation (3) is the edge weight of FWHVG. Equation (4) is the edge weight of BWHVG:

(3) $$w_{ij}^{f}=\arctan\frac{x_{j}-x_{i}}{t_{j}-t_{i}}+\arctan\frac{x_{i}}{t_{j}-t_{i}},t_{i}<t_{j},$$

(4) $$w_{ij}^{b}=\arctan\frac{x_{i}-x_{j}}{t_{j}-t_{i}}+\arctan\frac{x_{j}}{t_{j}-t_{i}},t_{i}<t_{j}$$

**Figure 4:**
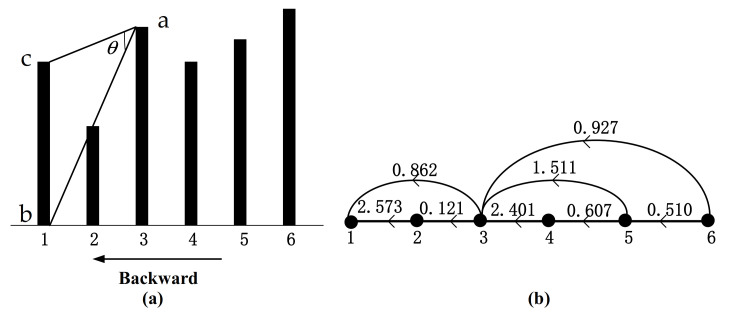
Graphical illustration of BWHVG of the time series. (**a**) Angle measurement of BWHVG; (**b**) Corresponding BWHVG of the time series.

The HVG algorithm is undirected, but the edge weight is related to the direction in our method. For a time series, when it is mapped forward to a weighted horizontal visibility graph, it can be named forward weighted horizontal visibility graph (FWHVG), as shown in Figure 3. When it is mapped back to a weighted horizontal visibility graph, it can be named backward weighted horizontal visibility graph (BWHVG), as shown in Figure 4. For a random time series given by *x* = {7.0,4.0,8.0,6.5,7.6,9.0}, the HVG can be found in Figure 2, and the graphical illustration of FWHVG and BWHVG can be found in Figure 3 and Figure 4. Figure 3a and Figure 4a show angle measurements of FWHVG and BWHVG between partial nodes. Figure 3b and Figure 4b show the networks mapped by FWHVG and BWHVG. Edge weights are different in different directed weighted horizontal visibility graphs.

The following example illustrates how edge weight is calculated. As it is clear from Figure 2 that *x*_1_ = 7.0 and *x*_3_ = 8.0 is visible. The angles between *x*_1_ and *x*_3_ of FWHVG and BWHVG are shown in Figure 3a and Figure 4a. The edge weight of FWHVG between the two nodes is:

(5) $$w_{23}^{f}=\arctan\frac{8.0-7.0}{3-1}+\arctan\frac{8.0-7.0}{3-1}\approx 1.756,$$

Thus, the edge weight between node 1 and node 3 is 1.756 in FWHVG. The weighted matrix of FWHVG can be calculated as:

$$W_{F}=\left[\begin{array}{cccccc} 1 & 0.180 & 1.756 & 0 & 0 & 0\\ 0.180 & 1 & 2.652 & 0 & 0 & 0\\ 1.756 & 2.652 & 1 & 0.467 & 1.123 & 1.534\\ 0 & 0 & 0.467 & 1 & 2.251 & 0\\ 0 & 0 & 1.123 & 2.251 & 1 & 2.391\\ 0 & 0 & 1.534 & 0 & 2.391 & 1 \end{array}\right]$$

The edge weight of BWHVG between the two nodes is:

(6) $$w_{23}^{b}=\arctan\frac{8}{3-1}+\arctan\frac{7.0-8.0}{3-1}\approx 0.862,$$

The edge weight between node 1 and node 3 is 0.862 in BWHVG. The weighted matrix of BWHVG can be calculated as:

$$W_{B}=\left[\begin{array}{cccccc} 1 & 2.573 & 0.862 & 0 & 0 & 0\\ 2.573 & 1 & 0.121 & 0 & 0 & 0\\ 0.862 & 0.121 & 1 & 2.401 & 1.511 & 0.927\\ 0 & 0 & 2.401 & 1 & 0.607 & 0\\ 0 & 0 & 1.511 & 0.607 & 1 & 0.510\\ 0 & 0 & 0.927 & 0 & 0.510 & 1 \end{array}\right]$$

### 3.4. Feature Extraction

The main objective of feature extraction is to obtain reliable data for emotion recognition. For this reason, time-domain features and complex network features are extracted from EEG data.

#### 3.4.1. Time-Domain Features

Nawaz et al. [41] compared different features in emotion recognition to identify the features that can effectively discriminate the emotions. Their study showed that the time-domain features are more suitable for emotion recognition compared with power, entropy, fractal dimension, and wavelet energy. However, the time-domain features have received less attention so far. In this paper, we will make a deep analysis of the validity of time-domain features for emotion recognition.

In the current study, six time-domain features are adapted from [41]. Suppose ${\left\{x\left(t\right)\right\}}_{t=1}^{N}$ represents an EEG signal with N data samples.

(1) Mean: Mean represents the average of the time series:
  (7) $$\bar{x}=\frac{1}{N}\sum_{t=1}^{N}x\left(t\right)$$
(2) Standard deviation: It represents the deviation of data compared with mean. The standard deviation is calculated as a square root of the average of the square of the difference between the EEG signal sample and the mean:
  (8) $$\sigma_{x}=\sqrt{\frac{1}{N}\sum_{t=1}^{N}\left(x(t)-\bar{x}\right)}$$
(3) First Difference: It represents the relationship between the current data and the previous data, and reflects the waveform dimensionality changes. First difference is calculated as the sum of the absolute difference between a pair of samples:
  (9) $$d_{st}=\frac{1}{N-1}\sum_{t=1}^{N-1}\left|x\left(t+1\right)-x\left(t\right)\right|$$
(4) Second Difference: It means the relationship between three adjacent data points and is a measure sensitive to the variation of the signal amplitude. The calculation of the second difference is similar to that of the first difference.
  (10) $$d_{nd}=\frac{1}{N-2}\sum_{t=1}^{N-2}\left|x\left(t+2\right)-x\left(t\right)\right|$$
  In following section, *X*(*t*) represents the normalized series as below:
  (11) $$X\left(t\right)=\frac{x\left(t\right)-\bar{x}}{\sigma_{x}}\left(t=1,2,\cdots ,N\right)$$
  where $\bar{x}$ and $\sigma_{x}$ can be found in Equations (7) and (8).
(5) First difference of normalized EEG: It is the relationship between the current data and the previous data of normalized EEG signal:
  (12) $$D_{st}=\frac{1}{N-1}\sum_{t=1}^{N-1}\left|X\left(t+1\right)-X\left(t\right)\right|$$
(6) Second difference of normalized EEG: It represents the relationship between three adjacent data points of normalized EEG signal:
  (13) $$D_{nd}=\frac{1}{N-2}\sum_{t=1}^{N-2}\left|X\left(t+2\right)-X\left(t\right)\right|$$

#### 3.4.2. Network Statistical Properties

The original series ${\left\{x\left(t\right)\right\}}_{t=1}^{N}$ is mapped into weighted networks. Then the network metrics can be extracted.

(7) Average weighted degree

In unweighted networks, the edge number of one node connected with other nodes is called degree. In general, the larger degree of the node, the greater importance of the network. In a weighted network, the weighted degree *d*_i_ can be extended to the strength of node *t_i_* [21]. The average weighted degree can be represented as Equation (15):

(14) $$d_{i}=\sum_{j=1}^{N}w_{ij}$$

(15) $$\bar{d}=\frac{1}{M}\sum_{i=1}^{N}d_{i}$$

where *w_ij_* is the edge weight between node *t_i_* and *t_j_*.

(8) Deviation of weighted degree

The deviation of weighted degree can be calculated as follows [42]:

(16) $$d_{std}={\left(\frac{\sum_{i=1}^{N}{\left(d_{i}-\bar{d}\right)}^{2}}{N-1}\right)}^{1/2}$$

(9) Weighted clustering coefficient

Clustering coefficient and clustering coefficient entropy [43] describes the relationship between one node and its neighbors. The weighted clustering coefficient of the network can be calculated from the average weighted clustering coefficient of all nodes in the network, as shown in Equation (17):

(17) $$C=\frac{1}{M}\sum_{i=1}^{N}C_{i}$$

(18) $$C_{i}=\frac{\sum_{j,k}w_{ij}w_{ik}w_{jk}}{\sum_{k\neq j}w_{ij}w_{ik}}$$

where *C_i_* is the weighted clustering coefficient of node *t_i_*, *w_ik_* is the weight between node *t_i_* and *t_k_*, *w_jk_* is the weight between node *t_j_* and *t_k_*, *w_ij_* is the weight between node *t_i_* and *t_j_*.

(10) Weighted clustering coefficient entropy

Weighted clustering coefficient entropy *E_C_* can be calculated as follows:

(19) $$E_{C}=-\sum_{j=1}^{N}P_{C,i}\log\left(P_{C,i}\right)$$

(20) $$P_{C,i}=C_{i}/\sum_{j=1}^{N}C_{i}$$

where *P_C,i_* is the probability of the weighted clustering coefficient of node *t_i_*.

### 3.5. Feature Fusion 

After extracting the features of complex networks, two kinds of visibility graph features are fused. The procedure can be described as follows:

(1) Setting a sliding time-window to divide the EEG signals into *M* segments.
(2) EEG segments are mapped to FWHVGs and complex network features are extracted. For a feature, we can get the feature vector $Y_{\mathrm{FWHVG}}=\left[y_{1}^{f},y_{2}^{f},\cdots ,y_{M}^{f}\right]$.
(3) Then we map EEG segments to BWHVGs, and extracted complex network features. For a feature, we get the feature vector $Y_{\mathrm{BWHVG}}=\left[y_{1}^{b},y_{2}^{b},\cdots ,y_{M}^{b}\right]$.
(4) Finally, the fusion feature vector is calculated as Equation (21):
  (21) $$\begin{array}{ll} G=Y_{\mathrm{FWHVG}}+Y_{\mathrm{BWHVG}} & =\left[y_{1}^{f},y_{2}^{f},\cdots ,y_{M}^{f}\right]+\left[y_{1}^{b},y_{2}^{b},\cdots ,y_{M}^{b}\right]\\ & =\left[g_{1}^{},g_{2}^{},\cdots ,g_{M}^{}\right] \end{array}$$
  where *g*_1_, *g*_2_, ..., *g_M_* is the element of G. $y_{1}^{f},y_{2}^{f},\cdots ,y_{M}^{f}$ and $y_{1}^{b},y_{2}^{b},\cdots ,y_{M}^{b}$ are the elements of $Y_{FWHVG}$ and $Y_{BWHVG}$, separately.

The values of different features may vary greatly, so it is necessary to normalize the features to reduce the difference. Mapping the feature vector between 0 and 1 to avoid the classification error caused by the large difference of features. The normalized result ${\bar{g}}_{m}$ is expressed by Equation (22):

(22) $${\bar{g}}_{m}=\frac{g_{m}-g_{\min}}{g_{\max}-g_{\min}},\left(m=1,2,\cdots ,M\right)$$

where $g_{m}$ is the element of **G**; $g_{\min}$ and $g_{\max}$ represent the maximum and minimum values of **G**. The normalized feature vector is $\bar{G}=\left({\bar{g}}_{1},{\bar{g}}_{2},\cdots ,{\bar{g}}_{M}\right)$. For four complex network features, the normalized feature matrix can be represented as ${\bar{G}}_{all}={\left({\bar{g}}_{im}\right)}_{4\times M}$. For six time-domain features, the normalized feature matrix is ${\bar{E}}_{all}={\left({\bar{e}}_{jm}\right)}_{6\times M}$. The normalized feature matrix of combined features can be represented as $\bar{Y}=\left[\begin{array}{c} {\bar{G}}_{all}\\ {\bar{E}}_{all} \end{array}\right]={\left({\bar{y}}_{nm}\right)}_{10\times M}$. 

### 3.6. Classification

Support vector machines (SVM), optimized fitted k-nearest neighbors (OF-KNN) and decision tree (DT) classifiers are used for classification in this part. Based on promising empirical results of the three classifiers, we used them for emotion classification [41,44,45,46]. Besides, in the Section 4.4.1, the effectiveness of different scenarios based on [41] were compared. We used the same classifiers as this reference. Complementary information from different classifiers may lead to higher accuracy.

#### 3.6.1. Support Vector Machines (SVM)

We use a library for support vector machines (LIBSVM) in our work. It is a further improvement made on the SVM [47]. LIBSVM can solve the two-class problem by constructing an optimal separating hyperplane. This hyperplane is linear, and the distance between the two groups is maximized. There are two important parameter, kernel function parameter *γ* and penalty factor *C*. Kernel function transfers the training samples into a higher dimensional feature space. penalty factor represents degree of penalty to misclassification of samples. *C* is 2 and *γ* is 1 in this paper. SVM is a small sample learning method with simple algorithm and good robustness. However, this algorithm is difficult to implement for large-scale training samples.

#### 3.6.2. Optimized Fitted K-Nearest Neighbors (OF-KNN)

KNN is a popular machine learning algorithm, which is very reliable for EEG data classification. KNN looks for a number *k* of samples (called k-neighbors) nearest to the incoming training sample and then predicts its class based on the most common class of its nearest neighbors [48]. The KNN classifier’s performance is mostly dependent on the choice of the distance parameter and the number of nearest neighbors *k*. In this paper, we used a variant of KNN called optimized fitted KNN. This algorithm can find hyperparameters that minimize five-fold cross validation loss by using automatic hyperparameter optimization. To pick the best estimate, the Bayesian optimization acquisition function ‘expected-improvement-plus’ is used. It calculates the best estimated feasible point using the ‘best-point’ function. This algorithm has high accuracy and is insensitive to outliers. However, when the sample is unbalanced, there will be a large prediction bias.

#### 3.6.3. Decision Tree (DT)

DT can change the complicated decision-making problems into simple processes with minimum computation time [49]. The advantages of the algorithm include that they are relatively easy to interpret and have good classification performance on many datasets. It performs the learning by splitting the input data into finer subgroups and assigning decision rules to the subgroups in model outputs. DT can produce feasible and effective results for large data sources in a relatively short time. It is not suitable for data with the strong correlation.

## 4. Results

### 4.1. Evaluation Metrics

Three classification metrics including accuracy (*Acc*), sensitivity (*Sen*), specificity (*Spe*), and precision (*Pre*) are used in this study [50].

(1) Accuracy

Accuracy is the most commonly used evaluated guideline. It represents the proportion of the sample that is classified correctly:

(23) $$Acc=\frac{TP+TN}{TP+TN+FP+FN}\times 100\%$$

(2) Sensitivity

Sensitivity, also called Recall, means the probability percentage that positive samples are classified as positive samples by the model:

(24) $$Sen=\frac{TP}{TP+FN}\times 100\%$$

(3) Specificity

Specificity means the probability of correctly classified negative instances:

(25) $$Spe=\frac{TN}{TN+FP}\times 100\%$$

(4) Precision

Precision refers to the probability of true positive to the positive determined by the model.

(26) $$Pre=\frac{TP}{TP+FP}\times 100\%$$

where *TP*, *TN*, *FP* and *FN* stand for true positive, true negative, false positive and false negative, respectively.

### 4.2. Preprocessing 

EEG signals are usually collected with noise in real life, which makes it challenging to design algorithms for emotion classification. EEG recording equipment may be affected by the surrounding environment. Muscle activity and eye movement can also bring the noise. The input signal used for emotion recognition should be the noise-filtered signal. The DEAP database provides a preprocessed version. The data has been down-sampled to 128 Hz, and a bandpass frequency filter from 4.0–45.0 Hz was applied in this version. We set a 10-s long sliding time-window with 50% overlap to divide the one-minute long EEG signals. Following this segmentation, a one-minute long EEG signal is divided into eleven 10-s long EEG segments. 

### 4.3. Analysis of Visibility Graph Networks

**Figure 5:**
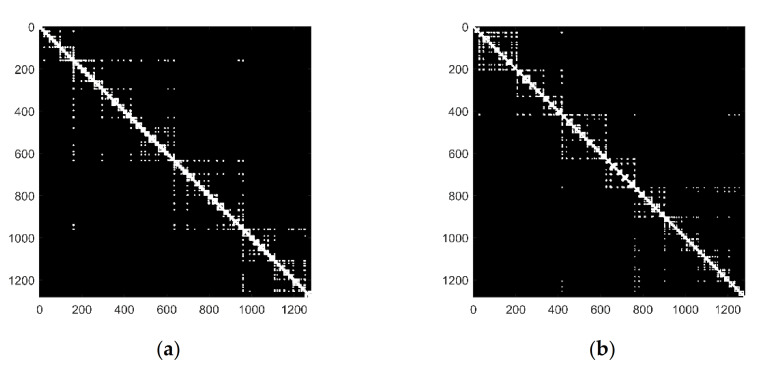
The adjacency matrix of networks based on VG. (**a**) EEG series with low valence; (**b**) EEG series with high valence.

**Figure 6:**
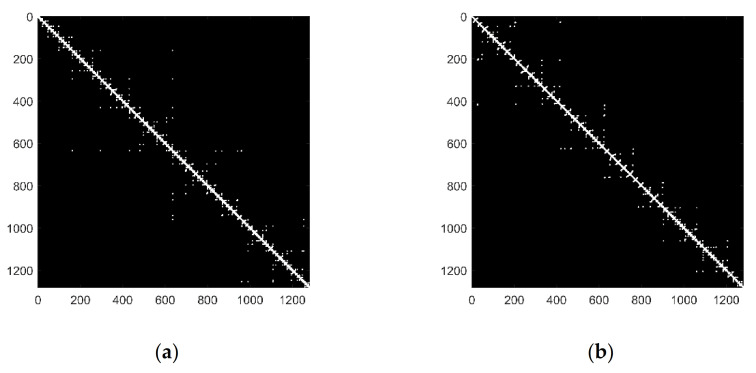
The adjacency matrix of networks based on HVG. (**a**) EEG series with low valence; (**b**) EEG series with high valence.

The emotion classes are assigned according to arousal and valence ratings done by subjects. It can be predetermined as two classes, i.e., low or high, based on the threshold of 5 on each dimension [51]. The labels are low valence and low arousal when the rating is less than 5. The labels are high valence and high arousal when the rating is greater than or equal to 5. The adjacency matrices of networks obtained from the EEG signal with high valence and low valence by applying VG are shown in Figure 5. As mentioned in Section 3.3.1, when a time series is mapped to an unweighted complex network, it can be expressed by an adjacent matrix. When two nodes are visible to each other, the value of the adjacent matrix is 1, otherwise, the value is 0. The white dots in Figure 5 and Figure 6 indicate the corresponding pair of nodes that are visible to each other, and the black portions represent no visibility. For each set of the data, 1280 samples were selected. The network connections of the EEG signal with low valence (Figure 5a) are tighter, and the clusters are much bigger. This indicates that its clustering characteristic is more obvious than the EEG signal with high valence (Figure 5b) in the control group.

The adjacency matrixes of networks based on the HVG method are shown in Figure 6. The information got from Figure 6 is similar to that in Figure 5. The network connections in Figure 6a are tighter compared with Figure 6b, and the clusters are much bigger. There are fewer white dots in Figure 6 than in Figure 5, which means that the number of connected edges in Figure 6 is less than that in Figure 5. This indicated that the network mapped by VG is more complicated than that mapped by HVG. From the above analysis, we can get that the visibility network is effective in emotion recognition. HVG retains part of the information in the VG. And its structure is more straightforward. So, the HVG method is chosen as the basis in our process. 

**Figure 7:**
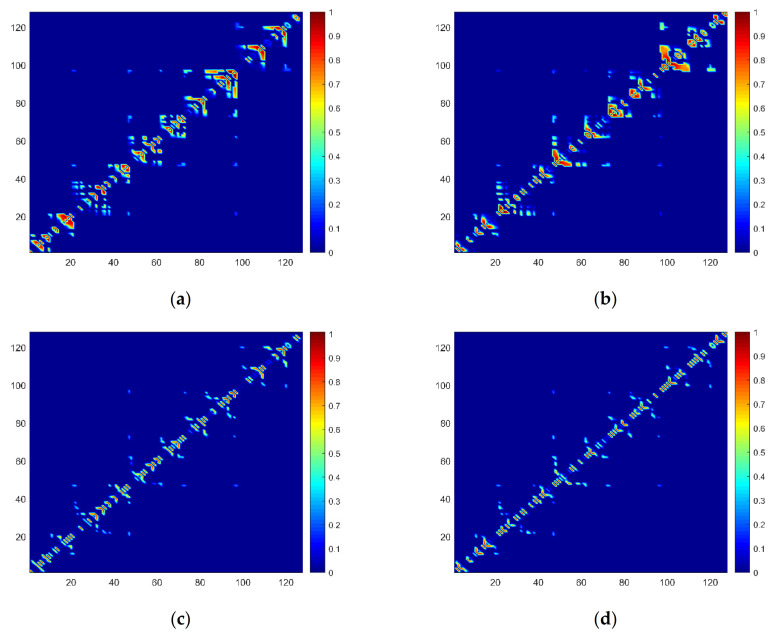
Visualization normalized weight matrices of forward and backward weighted complex networks. (**a**) FWVG; (**b**) BWVG; (**c**) FWHVG; (**d**) BWHVG.

Figure 7 shows a local refinement of weight matrices based on forward weighted complex networks and backward weighted complex networks. When a time series is mapped to a weighted complex network, it can be expressed by a weight matrix. The color represents the weighted edge, the larger the value, the darker the color. 128 samples were selected for easier comparison. The following four images are all from the same time series. The figures show the different edge weights of different methods. The weight matrices were normalized. Figure 7a,b are the weight matrices of the forward weighted visibility graph (FWVG) and backward weighted visibility graph (BWVG). Figure 7c,d are the weight matrices of the forward and backward weighted horizontal visibility graph. The edge weights of the elements nearby the diagonal part of the matrixes are much larger than those far away from the diagonal. In different graphs, elements with large weights are located in different places.

**Figure 8:**
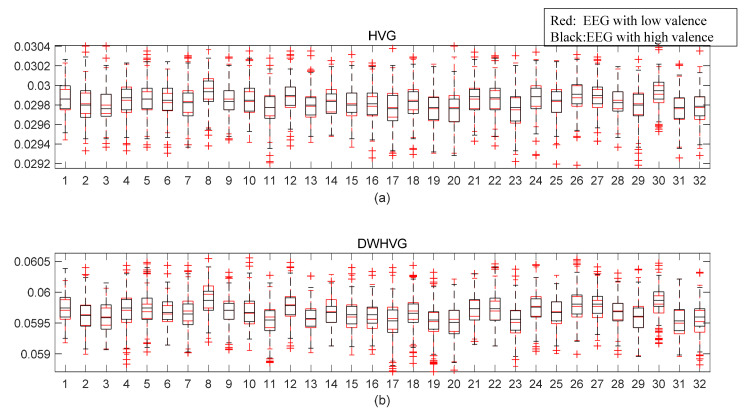
Box plots of the average weighted degree feature of 32 channels. (**a**) HVG; (**b**) DWHVG.

As mentioned above, 32 EEG channels are used to classify emotional states. That’s means, for a complex network feature, we can get 32-dimensional feature matrices. In this paper, four network properties were used for emotion recognition, as listed in Section 3.4.2. For one feature, the feature matrix of 32 EEG channels is 440 (segments) × 32 (channels). For four features, the feature matrix of 32 EEG channels is 440 (segments) × 128 (32 (channels) × 4 (features)). There was little difference in the classification results of the four features separately. Now, we randomly select a feature to compare the effectiveness of the different methods. The average weighted degree feature was selected here. Figure 8 shows box plots of the feature of 32 EEG channels based on HVG and DWHVG. The abscissa represents 32 EEG channels. Red box plots are the average weighted degree feature of EEG signals with low valence. Black box plots are the feature of EEG signals with high valence. It can be observed from the box plot that the differences in terms of median and quartiles in Figure 8b are more obvious than those in Figure 8a.

### 4.4. Classification Results

Five-fold cross validation and 10-fold cross validation were performed to evaluate participant’s samples and the mean of them was taken as the result of the subject. The average performance of all participants was calculated as the final results.

#### 4.4.1. Comparison of Time-Domain Features 

In [41], only 14-channels were selected for classifying emotional states. The selected EEG channels are located on AF3, F3, F7, FC5, T7, P7, O1, O2, P8, T8, FC6, F8, F4, and AF4. The first 20 s of data were excluded from EEG samples and the remaining 40-s long EEG signal was divided into four 10-s long segments without overlap. Grid searching was used to scan the available set of parameters for identifying the best parameter. The parameters of SVM and KNN were {*C*,*γ*} ∈ {10^−4^,10^−3^,10^−2^,10^−1^,1,10,10^2^,10^3^,10^4^,10^5^10^6^} and *k* ∈ {5,4,3,2,1}. To find out the effectiveness of different data lengths and the sliding window types, four scenarios were compared in this section. The same EEG channels and classifiers were used for classification. Five-fold cross validation was used as in [41].

*Scenario 1*: The plan used in [41].
*Scenario 2:* The remaining 40-s long EEG signal was divided by a 10-s long sliding time-window with 50% overlap.
*Scenario 3*: A 10-s long sliding time-window partitioned one-minute long EEG signal into six segments without overlap.
*Scenario 4*: One-minute long EEG signal was segmented by a 10-s long sliding time-window with 50% overlap.

In Scenario 1, there are 160 (40 (videos) × 4 (segments)) features for each participant on each channel. With 5-fold cross validation method, the numbers of training data and testing data are 128 and 32. In Scenario 2, 280 (40 (videos) × 7 (segments)) features are divided into five equal data with the number of 56. There are 240 (40 (videos) × 6 (segments)) features in Scenario 3. 5-fold cross validation method splits the data into 192 training data and 48 testing data. In Scenario 4, 440 (40 (videos) × 11 (segments)) features are divided into 352 training data and 88 testing data.

Average accuracies of the different scenarios for the valence and arousal classification tasks are presented in Table 2. When the sliding time window with an overlap rate of 50% is used for data segmentation, the classification accuracy is higher and the average sentiment recognition rates on 60-s long EEG signals are better than those on the remaining 40-s long EEG signal. In scenarios four, the classification accuracies are 95.68%, 94.60%, 85.19% for valence with SVM, KNN, and DT. The classification accuracies of arousal are 93.41%, 94.22%, 81.23%, respectively.

#### 4.4.2. Analysis of Complex Network Features 

In this section one-minute long EEG signal was divided by a 10-s long sliding time-window with 50% overlap. 32-channel EEGs were used for classifying emotional states. 10-fold cross validation method was used in following experiments. The performance estimation for complex network features of HVG and the proposed method are shown in Table 3 and Table 4.

As seen in Table 3 and Table 4, it is obvious that the OF-KNN method outperforms SVM and DT to classify valence and arousal. DT has the worst performance. With OF-KNN, we obtain the average classification accuracies for valence and arousal as 97.53% and 97.75% separately of proposed method. The performances of the HVG algorithm are 96.51% and 96.21% for valence and arousal. The classification accuracies of the proposed method in valence and arousal are respectively 1.02% and 1.24% higher than that of the HVG method. Most of the evaluation metrics in Table 3 are better than those in Table 4.

#### 4.4.3. Performance of Combined Features

In Section 4.4.1, only 14-channel EEGs were selected. In order to analyze the data more objectively, the remaining 18-channel EEG recordings were added for emotion recognition in this section, like Section 4.4.2. Table 5 shows the performance estimation for time-domain features of one-minute long EEG signals of 32 channels. Table 6 is listed the classification performance of combined features based on the proposed method and time-domain features. The combined features include time-domain features and complex network features of the proposed method.

The OF-KNN method is superior to SVM and DT in the classification of time-domain features and combined features. It has been observed from the results that the overall average accuracies of time-domain features are 97.78% and 97.37% under valence and arousal, with OF-KNN. Those of combined features are 98.12% and 98.06% separately. The classification accuracies of combined features in valence and arousal are respectively 0.42% and 0.69% higher than those of time-domain features, which are 0.59% and 0.31% higher than those of the proposed method (listed in Table 4). With OF-KNN, most the evaluation metrics of combined features are more stable compared with time-domain features. For example, in arousal dimensions, the STD of *Acc, Sen, Spe* and *Pre* based on time-domain features in the Table 5 are 2.35%, 3.85%, 2.37%, and 3.45%. Those of combined features in the Table 6 are 1.81%, 2.13%, 1.38% and 2.09%.

#### 4.4.4. Effectiveness of Different Classifiers

**Figure 9:**
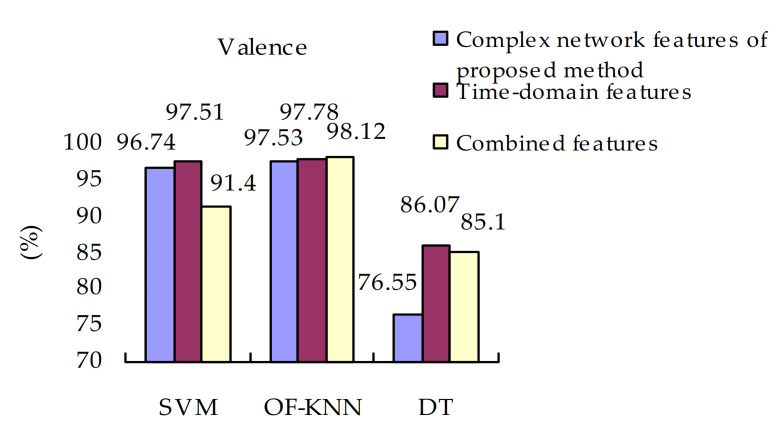
Classification accuracies of valence.

**Figure 10:**
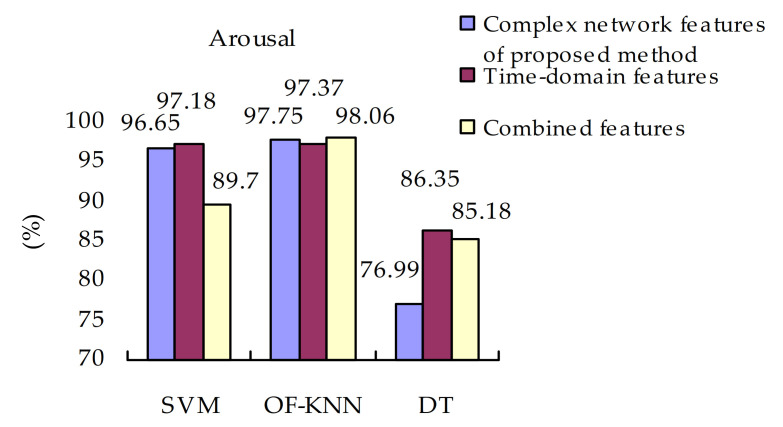
Classification accuracies of arousal.

The final experimental results for valence and arousal are shown in Figure 9 and Figure 10. The OF-KNN classifier can best distinguish EEG signals in valence and arousal dimensions than the other two types of classifiers. The emotion recognition method gets the lowest classification accuracy with the DT classifiers. When the SVM classifier is used, the classification accuracies of combined features are dropped compared with time-domain features and visibility graph features. The combination of the two types of features may not improve the classification accuracy. The evaluation metrics of OF-KNN is better than those in SVM and DT, and fluctuate less. The values of evaluation metrics of OF-KNN are smaller than those of SVM. But these metrics of SVM fluctuate a lot. This result partially reflects that the OF-KNN classifier outperformed SVM and DT in EEG-based emotion recognition in this paper.

## 5. Discussion

Many researchers have extracted features from EEG signals to identify the emotional state. Among these methods, time-domain features, entropy, and wavelet transform are widely used. In this study, we investigated the effectiveness of complex network metrics and time-domain features on emotion recognition.

For time-domain features, four scenarios were compared to find out the effectiveness of different data lengths and the sliding window types for emotion classification. The results showed that the method reached the highest accuracy when EEG signals were segmented by a 10-s long sliding time-window with 50% overlap. As mentioned above, each participant watches 40 one-minute long videos. At the same time, each participant has 40 one-minute long EEG recordings. As mentioned above, each participant watches 40 one-minute long videos. At the same time, each participant has 40 one-minute long EEG recordings. When six time-domain features are extracted from each channel, 192-dimensional (32 (channels) × 6 (features) = 192) feature matrices can be produced. 

In the case of complex network metrics, we constructed the DWHVG based on a new angle measurement method, in which the undirected network is relevant to the direction. EEG signals were mapped into FWHVGs and BWHVGs from different directions. On this basis, the fusion feature is used to improve the effectiveness of features. Extracting four network metrics on each channel of EEG data produces 128-dimensional (32 (channels) × 4 (features) = 128) feature matrices. It can be found that the proposed method is effective in recognizing emotion. 

SVM, OF-KNN, and DT classifiers were used for classification. The results reflected that the OF-KNN classifier outperformed SVM and DT in our method. The combination of the two types of features was fed into the three classifiers. Only OF-KNN shows a better classification rate. It is confirmed that the complex network features are effective in recognizing emotion. It provides a new research idea in emotion recognition.

The comparison of the proposed method with the existing methods is presented in Table 7. The emotion recognition problems in the references of Table 7 are all binary classification. The EEG signals used in the table all come from the DEAP database. Different feature extraction methods were compared in [41]. With the KNN classifier, the time-domain statistical characteristics achieved accuracies of 77.62% and 78.96% for valence and arousal respectively. Gao et al. [52] proposed a channel-fused dense convolutional network (CNN) for EEG-based emotion recognition. The deep-learning framework can obtain recognition accuracies over 92% for both valence and arousal classification tasks. Cui et al. [53] used an end-to-end regional-asymmetric convolutional neural network (RACNN) to reach accuracies of 96.65% and 97.11% under valance and arousal. An emotion recognition system transforming 1D chain-like EEG vector sequences into 2D mesh-like matrix sequences was proposed in [54]. The experimental results demonstrated that the classification accuracies of hybrid neural networks achieved 93.64% and 93.26% in valence and arousal dimensions. According to Liu et al. [55], a multi-level features guided capsule network (MLF-CapsNet) was used. A one-second long sliding time window divided the one-minute long EEG signal into 60 segments. The maximum recognition rates on valence and arousal were separately 97.97% and 98.31%. When combined with time-domain features, the proposed method showed the accuracies of 98.12% and 98.06% for valence and arousal.

According to values of arousal and valence, emotion states can also be divided into 4 types, high arousal high valence (HAHV), high arousal low valence (HALV), low arousal high valence (LAHV), and low arousal low valence (LALV). Zhang et al. [45] employed an empirical mode decomposition (EMD) strategy to decompose EEG signals, and then calculated corresponding sample entropies of the first 4 intrinsic mode functions (IMFs). The average accuracy for the 4-class task was 93.20%. Nonlinear features were extracted from EEG signals, and a feature selection method was used to enhance the classification performance [26]. MLP, KNN, and SVM combined through the voting algorithm as a combined classifier. A classification rate of 84.56% was achieved on the DEAP dataset, 90% on their dataset. The highest classification accuracy achieved by ANN for 4-class emotion, entropy-based features, and implementation is 93.75% in [56].

The limitations of this study are as follows. The preprocessed dataset provided by the DEAP database was used in this paper. We didn’t take into account the effect of noise. A study on noise robustness should be considered in future work. What more, the proposed method is only verified in the DEAP dataset, it should be performed and experimented with in different datasets. Besides, only two-level classification experiments of valence and arousal were considered in this paper. The multi-classification problem should be taken into consideration.

## 6. Conclusions 

This paper proposed a novel method based on an improved visibility graph network to recognize the emotion model, which classified the two emotional dimensions of arousal and valance. In this model, a weighted visibility graph construction method based on visibility angle measurement transforms an undirected network into a directed network. Then, the feature matrices extracted from different directions based on DHVG were integrated into new feature matrices through feature fusion. 

Thirty-two channel recordings of EEG signals were used in this implementation. Besides, we also extracted the time domain features. Three different machine learning classifiers were used to compare the feature extraction methods, which were SVM, OF-KNN, and DT. 

In the valence and arousal domain, the average emotion recognition rates based on complex network features of our proposed method achieved 97.53% and 97.75% with 10-fold cross validation. When combined with time-domain features, the average accuracies reached 98.12% and 98.06%. It is confirmed that the proposed method is effective in recognizing emotion.

In the process of emotion recognition, the combinations of different channels have different recognition results. In the future, we will explore how to use fewer EEG channels to achieve higher classification accuracy. Moreover, the multi-category of the emotional dimension is also worth studying.

## Figures and Tables

**Table 1:** Description of participant ratings part in DEAP dataset.

Materials	Setup	
Number of participants	32	
Number of videos	40	
Rating scales	Valence	Indicator of pleasantness (float between 1 and 9).
	Arousal	Measure of the intensity of the emotion (float between 1 and 9).
	Dominance	Feeling of being in control of the emotion (float between 1 and 9).
	Liking	Liking of the video (float between 1 and 9).
	Familiarity	Familiarity with videos (integer between 1 and 5). Blank if missing.
Recordings	32 EEG channels +8 other peripheral channels	

**Table 2:** Comparison of average sentiment recognition rates (%) on different scenarios.

	Valence				Arousal			
**Classifiers**	**S1**	**S2**	**S3**	**S4**	**S1**	**S2**	**S3**	**S4**
SVM	77.62	94.43	88.13	95.68	78.96	93.64	85.45	93.41
KNN	75.06	92.57	87.17	94.60	74.71	90.57	83.36	94.22
DT	71.48	82.59	77.15	85.19	72.93	81.40	75.04	81.23

**Table 3:** Performance estimation for complex network features of HVG (%).

		Valence			Arousal		
		SVM	OF-KNN	DT	SVM	OF-KNN	DT
*Acc*	Mean	80.17	96.51	64.84	80.38	96.21	66.39
	STD	3.82	0.80	4.57	4.14	1.48	5.78
*Sen*	Mean	69.19	98.42	59.55	63.04	97.67	57.15
	STD	20.23	1.32	9.61	27.80	2.46	13.96
*Spe*	Mean	84.65	98.60	66.99	80.69	97.24	66.31
	STD	11.28	1.02	7.76	21.92	2.44	13.94
*Pre*	Mean	82.42	98.32	59.38	82.88	97.75	56.91
	STD	6.57	0.84	9.22	8.48	2.13	14.09

**Table 4:** Performance estimation for complex network features of proposed method (%).

		Valence			Arousal		
		SVM	OF-KNN	DT	SVM	OF-KNN	DT
*Acc*	Mean	96.74	97.53	76.55	96.65	97.75	76.99
	STD	2.16	2.44	3.87	2.12	2.21	4.85
*Sen*	Mean	95.11	97.51	72.67	93.45	96.27	69.71
	STD	3.82	2.32	6.57	6.02	2.87	10.43
*Spe*	Mean	97.44	97.43	78.36	96.89	97.77	77.84
	STD	2.22	2.06	5.71	2.61	2.14	8.53
*Pre*	Mean	97.53	97.34	72.80	97.32	96.27	69.63
	STD	2.45	2.34	6.75	2.46	2.82	10.74

**Table 5:** Performance estimation for time-domain features (%).

		Valence			Arousal		
		SVM	OF-KNN	DT	SVM	OF-KNN	DT
*Acc*	Mean	97.51	97.78	86.07	97.18	97.37	86.35
	STD	1.77	1.79	3.40	2.13	2.35	4.08
*Sen*	Mean	95.87	97.70	84.22	94.16	96.63	82.25
	STD	2.72	2.09	4.65	5.83	3.85	7.22
*Spe*	Mean	98.43	97.79	86.67	96.66	97.47	87.04
	STD	1.48	1.70	4.73	3.80	2.37	5.6
*Pre*	Mean	98.47	97.60	84.08	98.10	96.45	82.03
	STD	1.07	1.74	4.28	1.47	3.45	6.80

**Table 6:** Performance estimation for combined features (%).

		Valence			Arousal		
		SVM	OF-KNN	DT	SVM	OF-KNN	DT
*Acc*	Mean	91.40	98.12	85.10	89.70	98.06	85.18
	STD	4.36	1.79	4.02	4.69	1.81	4.20
*Sen*	Mean	81.52	97.98	83.13	74.85	97.44	80.54
	STD	17.70	1.86	4.46	23.04	2.13	7.52
*Spe*	Mean	92.24	97.14	85.93	82.03	98.12	85.74
	STD	6.69	2.21	5.30	15.00	1.38	6.12
*Pre*	Mean	94.24	97.97	83.04	93.84	97.51	80.74
	STD	4.58	1.63	4.87	5.94	2.09	6.81

**Table 7:** Comparison of the proposed work with previous works (%).

Methods	Input	Number of Channels	Length of Signal	Classifier	Average Accuracy	
					Valence	Arousal
[41]	Time domain features	14	40s	KNN	77.62	78.96
[52]	DE features	32	60s	Dense CNN	92.24	92.92
[53]	Spatial encoding of EEG signals	32	60s	RACNN	96.65	97.11
[54]	2D PSD mesh sequence	32	60s	CNN-RNN	93.64	93.26
[55]	Raw EEG signals	32	60s	MLF-CapsNet	97.97	98.31
Proposed method	Complex network features	32	60s	OF-KNN	97.53	97.75
Proposed method	Complex network features + Time-domain features	32	60s	OF-KNN	98.12	98.06

## Data Availability

Publicly available dataset was analyzed in this study. This data can be found here: http://www.eecs.qmul.ac.uk/mmv/datasets/deap/ (accessed on 22 February 2021).
